# Personal

**Published:** 1889-06

**Authors:** 


### Personal.

Dr. J. B. Andrews, Superintendent Buffalo State Asylum for the Insane, sailed for Europe on May 23, 1889, for three months' vacation. He will visit Great Britain and the Continent, spending some time with distinguished alienists abroad.

Dr. R. Stansbury Sutton, of Pittsburgh, Pa., conducts one of the best-appointed and most successful private hospitals for women in the country. He has recently completed a series of fifty abdominal sections for ovarian cysts and otherwise diseased ovaries and tubes, with but three deaths.

Dr. Roswell Park, of this city, has been invited to deliver the Mütter lectures on "Some Point or Points Connected with Surgical Pathology," before the Philadelphia College of Physicians, during the Winter of 1890-91.

Dr. J. M. Matthews, of Louisville, whose trenchant pen is familiar to readers of medical periodical literature, will, it is announced, resume editorial work upon that bright and able journal, *Progress*. As both Reynolds and Matthews can "read readin' and write writin'," great results are likely to ensue from this combination of talent. When these two pull together, "suthin' has to give."

---

DR. A. A. HUBBELL, President of the Buffalo Medical and Surgical Association, delivered an interesting address on the Retrospective and Prospective Conditions of that much neglected and somewhat abused, though still honorable, medical Society. We should like to print the address had we the space at command, for it would make interesting reading.

---

DR. WILLIAM C. KRAUSS, of Attica, N. Y., who has been several years in Europe pursuing medical studies, sailed homeward from Hamburg, May 26th ult. Dr. Krauss took his medical degree at the Berlin University last year, and has been a frequent contributor to the columns of this journal, from his Parisian residence during the past several months. We hope to see him in Buffalo early in June.

---

DRS. GEORGE S. PECK and A. M. Clark, of Youngstown, O., sailed for Europe by the North German Lloyd steamer "Ems," Saturday, May 25, 1889. They will remain abroad for six months in observation and study of medical and surgical work in the medical centers of Great Britain and the Continent.

---

DR. A. E. PERSONS, Professor of Materia Medica and Therapeutics, Medical Department of Niagara University, has removed from No. 104 William street to No. 93 West Mohawk street.

